# Premature aortic smooth muscle cell differentiation contributes to matrix dysregulation in Marfan Syndrome

**DOI:** 10.1371/journal.pone.0186603

**Published:** 2017-10-17

**Authors:** Matthew Dale, Matthew P. Fitzgerald, Zhibo Liu, Trevor Meisinger, Andrew Karpisek, Laura N. Purcell, Jeffrey S. Carson, Paul Harding, Haili Lang, Panagiotis Koutakis, Rishi Batra, Constance J. Mietus, George Casale, Iraklis Pipinos, B. Timothy Baxter, Wanfen Xiong

**Affiliations:** Department of Surgery, University of Nebraska Medical Center, Omaha, Nebraska, United States of America; Universidade de São Paulo, BRAZIL

## Abstract

Thoracic aortic aneurysm and dissection are life-threatening complications of Marfan syndrome (MFS). Studies of human and mouse aortic samples from late stage MFS demonstrate increased TGF-β activation/signaling and diffuse matrix changes. However, the role of the aortic smooth muscle cell (SMC) phenotype in early aneurysm formation in MFS has yet to be fully elucidated. As our objective, we investigated whether an altered aortic SMC phenotype plays a role in aneurysm formation in MFS. We describe previously unrecognized concordant findings in the aortas of a murine model of MFS, mgR, during a critical and dynamic phase of early development. Using Western blot, gelatin zymography, and histological analysis, we demonstrated that at postnatal day (PD) 7, before aortic TGF-β levels are increased, there is elastic fiber fragmentation/disorganization and increased levels of MMP-2 and MMP-9. Compared to wild type (WT) littermates, aortic SMCs in mgR mice express higher levels of contractile proteins suggesting a switch to a more mature contractile phenotype. In addition, tropoelastin levels are decreased in mgR mice, a finding consistent with a premature switch to a contractile phenotype. Proliferation assays indicate a decrease in the proliferation rate of mgR cultured SMCs compared to WT SMCs. KLF4, a regulator of smooth muscle cell phenotype, was decreased in aortic tissue of mgR mice. Finally, overexpression of KLF4 partially reversed this phenotypic change in the Marfan SMCs. This study indicates that an early phenotypic switch appears to be associated with initiation of important metabolic changes in SMCs that contribute to subsequent pathology in MFS.

## Introduction

Marfan syndrome (MFS) is an autosomal dominant inherited disorder of connective tissue with prominent abnormalities in the cardiovascular, skeletal, and ocular systems. It is caused by mutations in *FBN1*, the gene encoding fibrillin-1 [1, 2]. The leading cause of mortality in patients with MFS is progressive aortic root dilatation, aneurysm formation and aortic dissection [3]. Histopathological features of aneurysm tissues in MFS include elastic lamellae fragmentation and disorganization, accumulation of amorphous matrix components, excessive smooth muscle cell (SMC) apoptosis, and increased expression of matrix metalloproteinase (MMP)-2 and MMP-9 [4–6]. However, it is unclear which, if any, of these late findings are causally related to aneurysm formation.

The vascular smooth muscle cells (VSMCs) are the main cell type within the media; they are uniquely responsible for synthesis of matrix proteins during development or in response to injury. They play a pivotal role in angiogenesis and vasculogenesis during embryonic development. After the initial differentiation of aortic precursor cells to a SMC lineage, VSMCs express SM-marker genes and proliferate rapidly [7–9]. Growth and repair of aorta are possible because VSMCs are capable of altering their phenotype allowing migration, proliferation, and/or elaboration of extracellular matrix. The VSMC phenotype is typically characterized by expression of smooth muscle contractile genes such as SM22α, α-SM-actin, calponin, and SM-myosin heavy chain (SM-MHC). They are more abundantly expressed in the contractile VSMCs and reduced in synthetic VSMCs [10]. Knowledge of the key role of SMC in these processes are informed by a variety of human SMC-linked diseases associated with altered expression of these contractile proteins [11, 12]

Studies using mouse models of MFS have implicated enhanced transforming growth factor (TGF)-β activation and signaling in the progression of numerous manifestations of the disease including aortic aneurysms [13–16]. TGF-β exerts a broad range of biological activities that impact cell proliferation, differentiation, and apoptosis during normal growth and development [17, 18]. The effects of TGF-β on the phenotype of VSMCs have been widely investigated. However, the role of TGF-β activation in mgR mice during early development is not entirely understood. In the present study, we demonstrate that active TGF-β levels are not increased in the early postnatal period, PD7, in mice with Marfan syndrome.

In a previous study, we demonstrated that doxycycline and losartan exhibit protective effects on matrix and delay aneurysm formation and rupture in mgR mice [6]. However, this treatment was only effective when started by PD1. Doxycycline failed to exhibit any protective effect when begun on PD21, suggesting that pathological changes were occurring in the early postnatal period. Aortic development occurs between embryonic day (ED) 14 and PD14 accompanied by important changes in hemodynamics and growth between birth and PD14 [19]. We hypothesized that the VSMC population in MFS undergoes a phenotypic change in response to the abnormal elastic fibers that impacts disease progression. We analyzed the VSMC phenotypes in mgR mice. Prior to an increase in active TGF-β levels, we find clear differences in the expression of VSMC markers, including KLF4 which has been shown to regulate SMC phenotype. In addition, we demonstrate that KLF4 overexpression in SMC from Marfan aorta can partially reverse these phenotypic changes.

## Materials and methods

### Mice

Heterozygous mutant mice (*Fbn1*^*mgR/+*^ or mgR/+) in a mixed C57BL/6J;129 SvEv background were mated to generate homozygous *Fbn1* mutant mice (*Fbn1*^*mgR/mgR*^) (also known as mgR) and wild type (WT) littermates [20]. Genotyping of mice was performed at PD7 by PCR [20, 21]. A group of WT littermates (n = 18) and a group of homozygous mgR mice (n = 18) were sacrificed by an approved institutional ethics committee protocol using anesthesia and exsaguination at PD7 to extract aortic tissue. Because of the small size of the mouse on PD7, this was the earliest time point where we were able to extract an adequate amount of protein for Western blot analysis from individual aortas. The ascending thoracic aortas were perfusion-fixed with 10% neutral buffered formalin and collected for histological studies. One-third of the samples were snap frozen in liquid nitrogen for protein extraction. For RNA studies, aortic samples were harvested and submerged in RNA*later* solution (Life Technologies). Thoracic aortic tissue from the heart to the descending aorta was harvested after mice were anesthetized. All experiments were carried out in accordance with the guidelines of the University of Nebraska Medical Center Animal Care Committee for the use and care of laboratory animals (approved IACUC protocol permit #94-003-10-FC). All mice were maintained in the pathogen free animal facility. The UNMC animal care facility is registered with the United States Department of Agriculture under the Animal Welfare Act and is accredited by AAALAC International. UNMC IACUC and animal care accreditations: Assurance #A3294-01 and AAALAC Accreditation #00031. The care and use of animals for research at UNMC is based on Federal Regulations.

**Fig 1:**
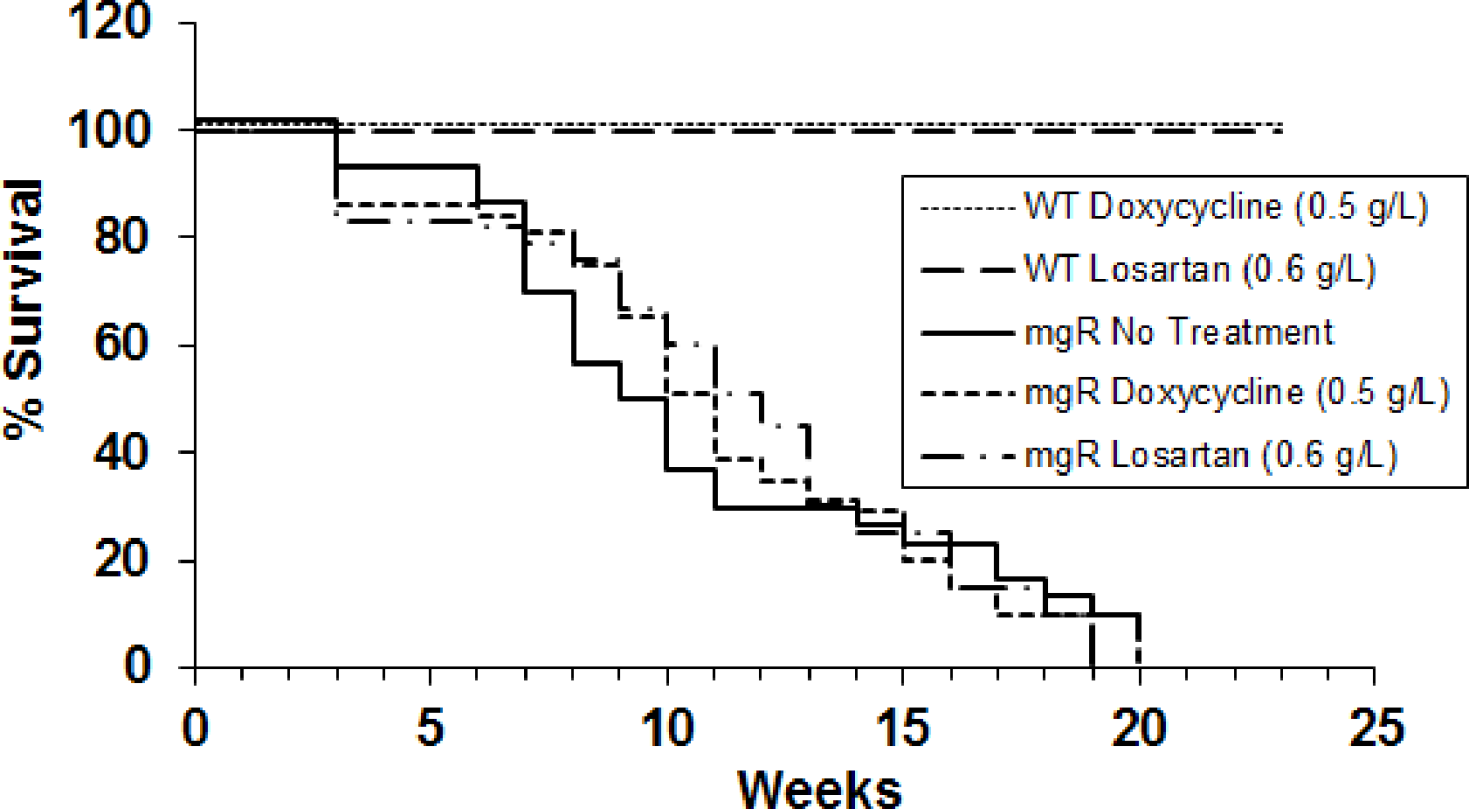
The survival of mgR mice with treatment of doxycycline and losartan started at PD21. MgR mice were untreated or treated with doxycycline (0.5 g/L) or losartan (0.6 g/L) in their drinking water started at PD21. Life table analysis shows that both treatments are not effective on survival of mgR mice compared with untreated mice if treatments started at PD21.

Humane treatment of animals: the Marfan mouse model (mgR) used in these studies produce 25% of normal fibrillin production. They form aortic aneurysms and mice die of aortic rupture between 8–12 weeks of age. These mice show no signs of distress or pain until aortic rupture, and the dosage levels used of losartan and doxycycline in the experiments in Fig 1 have been shown to be safe and non-toxic [15, 20]. Therefore, no administration of pain relievers are necessary for this animal model. However, throughout these studies mice were monitored daily for humane endpoints by the following criteria as approved by IACUC protocol: behaviour changes, adverse reactions to losartan or doxycycline treatments, eating and drinking, grooming, posture, activity level, ambulation, and overall wellness. Study mice were monitored by both trained study staff and by vet techs at UNMC animal care facility. No animals during the duration of this study needed to be euthanized due to drug reactions or changes in wellness as outlined in the approved criteria above. All animal studies and mortality aspects of these experiments were approved by the University of Nebraska Medical Center Animal Care Committee.

### Doxycycline and losartan treatment and Kaplan-Meier’s survival curve

Beginning on postnatal day (PD)21, mgR were given plain water, doxycycline (0.5 g/L) (Sigma, St. Louis, MO), or losartan (0.6 g/L)(AK Scientific, Inc.) in their drinking water (n = 15 mice per group for total of 45 mice). Water bottles containing doxycycline were covered with foil. As littermate controls we treated WT mice with doxycycline (0.5 g/L) (n = 5) and losartan (0.6 g/L) (n = 4). The concentration of doxycycline and losartan was chosen based on previous studies of kinetics, safety and treatment effects in mice [15, 20]. To configure the Kaplan-Meier survival curves, Fbn1^mgR/mgR^ mice with and without treatment were evaluated daily and survival recorded. Mice were monitored daily for any adverse reactions and followed up to 23 weeks. All animal mgR animals (n = 45) died from ruptured aortic aneurysms, while WT treated mice showed no signs of drug toxicity or shortened lifespan (n = 9). Mice were not euthanized prior to death using humane endpoints because they showed no signs of distress, adverse drug reaction or behavioural changes as outlined in our approved IACUC protocol. The mgR mice mimic human Marfan Syndrome with an acute rupture of aorta, therefore, these mice remain healthy and active until aortic rupture. The mortality aspects of this study were approved by the University of Nebraska Medical Center Animal Care Committee.

### Verhoeff-Van Gieson connective tissue staining

After perfusion-fixation with 10% neutral-buffered formalin, mouse ascending aortic tissues were embedded in paraffin and cut into 4-μm sections. The slides were stained with Verhoeff’s solution, ferric chloride, sodium thiosulfate, and Van Gieson’s solution (Poly Scientific). Each staining cycle alternated between fixing and washing procedures [22]. The slides were examined and photographed using light microscopy (×40; Nikon).

### Immunofluorescence and Immunohisctochemical staining

Paraffin embedded mouse aortic sections were cleared twice in 100% xylene and rehydrated through a series of ethanols (100%, 95%, 70%, and 50%). Sections were then washed with 1× PBS (pH 7.4) and blocked with 10% normal goat serum in PBS for 1 h at room temperature. Tissue sections were subsequently incubated with anti-calponin antibody (Santa Cruz Biotechnology, sc-28545) for 1 h at room temperature. After 3 washes in PBS, sections were incubated with isotype specific Alexa Fluor 488 (Invitrogen) for 1 h at room temperature. After 3 washes in PBS, the coverslips were mounted with Prolong Gold anti-fading reagent with DAPI (Invitrogen). Fluorescence images were captured with a Leica epifluorescence wide-filed microscope (10X objective) (North Central Instruments DMRXA2 Model) and CCD camera (Hamamatsu Photonics), with Hamamatsu software (HCImage 4.0). Grey scale images were generated for analysis with Image-Pro® Plus (Media Cybernetics, Bethesda, MD, USA). Calponin positive events in the aortae were partitioned and both event area and mean pixel intensity were determined for each (12-bit scale). The total area of the cross-section of each aorta, areas of positive events per cross-section, mean pixel intensities of positive events, and product of percent area occupied by positive events and mean intensity of the positive events (relative abundance of calponin per cross-section of aorta) were computed per microscopic field.

Immunohistochemical staining was performed with Ki-67 antibody (Abcam, ab16667) and α-actin (Abcam, ab5694) with a dilution of 1:200 and 1:1600 respectively. Discovery ChromoMap DAB kit (Roche, Cat: 760–159) was used for antigen localization and the slides were processed using Ventana Discovery Ultra instruments (Roche). Microscopic fields (40x objective) of aortae were collected from each slide labeled ***[with primary antibody*, *DAB converted HRP secondary antibody*, *and hematoxylin]***. Images were captured by multispectral wide-field microscopy, with a Leica microscope (North Central Instruments DMRXA2 Model) coupled with the Nuance EX Multispectral Imaging System (PerkinElmer N-MSI-EX Model) that incorporates a CCD camera and liquid crystal tunable filter. This system generates an absorbance spectrum at each pixel of a two-dimensional spatial image of the specimen. The Nuance software quantitatively extracts the grey scale image of deposited DAB and hematoxylin labeling, which is then transferred to the Image-Pro® Plus image analysis software (Media Cybernetics) for quantification of staining area and density. The area-weighted mean density of αSMA was determined for five regions of interest per aorta, as the sum of the products of area and mean pixel intensity of all positive events per microscopic field divided by the sum of areas of positive events (12-bit grey scale).

### Isolation of mouse SMC and cell culture

WT and their littermate mgR mice at 7 days old were anesthetized and underwent thoracotomy. Mouse thoracic aortas were isolated and minced. SMC isolation was described previously [22]. The cells were grown to confluence and passed after trypsinization with 0.25% trypsin. Mouse SMC were maintained using the Vascular Smooth Muscle Cell Growth Kit (ATCC). Experiments were performed on cells from passage 2–4.

### Cell proliferation assay

Aortic SMCs on the second passage were seeded into 96-well plates at density of 2500 cells/well for 24 h to serum-starvation. After 24 h culture in serum-free medium, AlamarBlue reagent (Invitrogen) was added to the cells to assess proliferation according to the manufacturer’s protocol. Absorbance values were measured by spectrophotometry using a Microplate Reader (Bio-Rad Laboratories, Inc.) at 570 and 600 nm at 3, 18, and 24 h after AlamarBlue was added. The relative cell viability was determined by calculated values of the percent difference in reduction of AlamarBlue between WT and mgR aortic SMC as described in the manufacturer’s instruction. The experiments were carried out in six replicates. For EdU staining: Aortic SMC (10^6^ cells) were suspended in F12K medium and incubated with 10 μmol/L EdU (Invitrogen) for 2 h. For a negative staining control, cells from the same population were not treated with EdU. After incubating with EdU, cells were washed with 1% BSA in PBS twice and incubated with 0.5 ml of Click-iT reaction cocktail for 30 minutes according to manufacturer’s instruction. This same EDU protocol was also carried out on aortic mgR SMCs with either empty vector (AdEmpty) or KLF4 (AdKLF4) adenovirus infection after 48 hrs. Cells were analyzed using a flow cytometer (BD Biosciences).

### Western blot analysis and gelatin zymography

Aortic proteins were extracted as previously described [22]. The protein concentration of aortic proteins and SMC cell extracts was standardized with a Bio-Rad protein assay. Equal amounts (25 μg) of aortic tissue extracts or aortic SMC extract from WT and mgR mice were loaded under reducing conditions onto a 10% SDS-polyacrylamide gel and transferred to a polyvinylidene difluoride (PVDF) membrane (Amersham Biosciences) The membranes were then incubated with the following primary antibodies: TGF-β (Cell Signaling 3711S), KLF4 (Cell Signaling 4038S), α-actin (Santa Cruz Biotechnology, (1A4) sc-32251), Tropoelastin (Elastin Products Co. PR385), MYH11 (Santa Cruz Biotechnology, (H-44) sc-98705, Calponin (Santa Cruz Biotechnology, (FL-297) sc-28545), SM22α (Santa Cruz Biotechnology (H-75) sc-50446), β –actin (Cell Signaling 4967L) and GAPDH (Cell Signaling 5174S). The bound primary antibody was detected with HRP-linked anti-mouse or anti-rabbit IgG (Cell Signaling 7076S and 7074S). Immunoreactive bands were visualized by autoradiography using ECL (Amersham Biosciences). Gelatin zymography for aortic tissue extract and SMC conditioned media was performed as described previously by Longo et al. [23], with 0.8% gelatin in a 10% SDS-polyacrylamide gel. The molecular sizes were determined using protein standards (Fermentas).

### RT-PCR assays for gene expression

All RNA from aortic tissues and SMCs were extracted using TRIzol reagent (Thermo Fisher Scientific). RNA was reverse transcribed into cDNA using iScript Reverse Transcription Supermix (Bio-Rad Laboratories, Inc.). Real-time RT-PCR was performed using SsoAdvanced Universal SYBR® Green Supermix according to the manufacturer’s instruction (Bio-Rad Laboratories,Inc.) on an ABI StepOne machine (Thermo Fisher Scientific). Fold differences were calculated using mRNA expression normalized to 18s and analyzed using the ΔΔCt relative quantification method.

### Adenoviral overexpression of KLF4

Overexpression of mouse KLF4 was achieved by adenovirus infection (AdKLF4) or an empty vector control (AdEmpty) in Aortic mgR SMC in the second passage with a multiplicity of infection (MOI) of 25 for 48h. Adenoviruses were purchased from Vector BioLabs, Malvern, PA.

### Statistical analyses

Data are presented as mean ± SE. Life table analysis was used for the Kaplan-Meier survival curve. Data were assumed to be nonparametric and analysis of variance (ANOVA) was used for comparisons. Kruskal-Wallis One-Way ANOVA on Ranks was used for statistical analysis of IF and IHC quantification experiments.

## Results

### Aortic development is altered by PD7 in mgR mice

**Fig 2:**
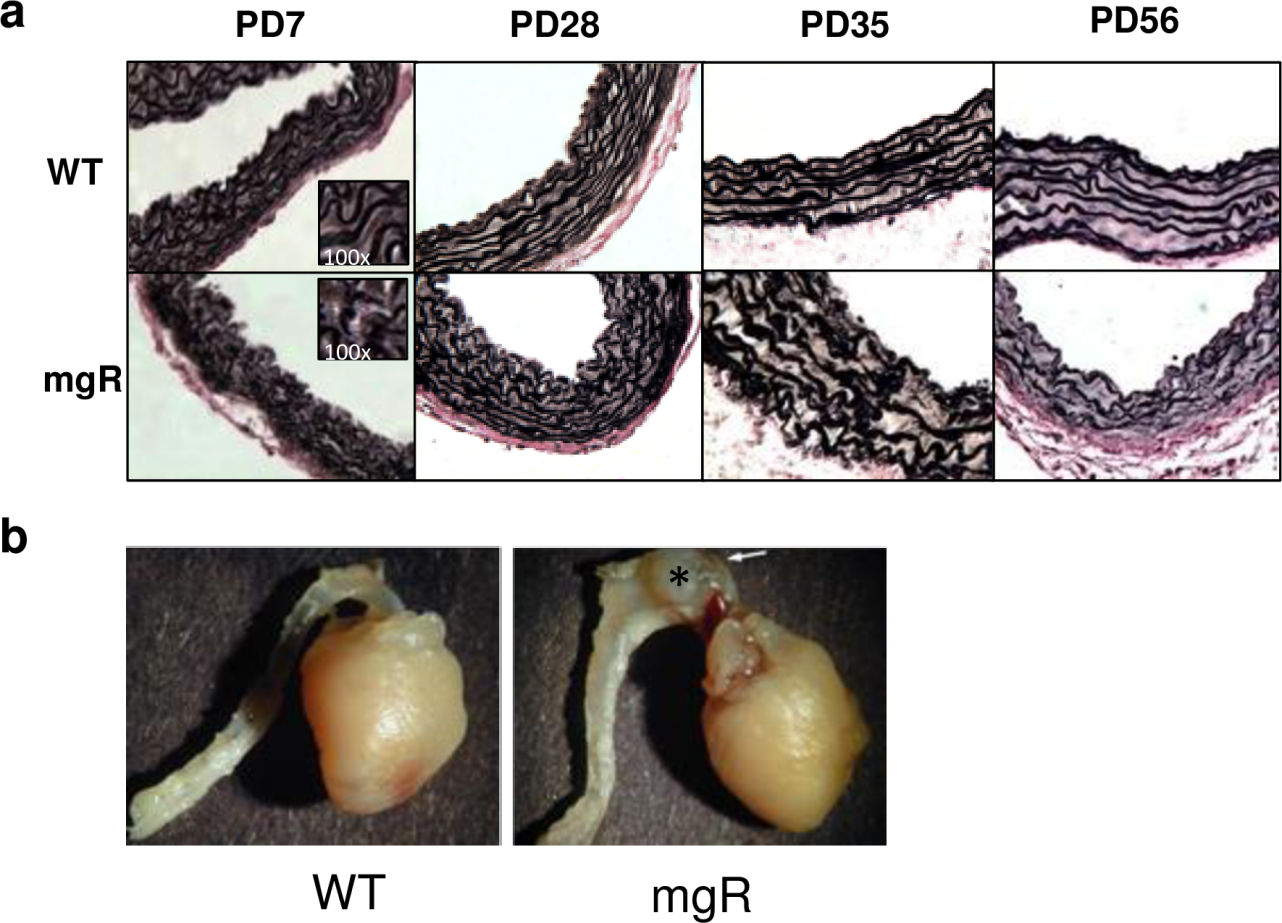
Progressive elastic fiber degradation and fragmentation with age. a)Verhoeff-van Gieson staining of elastic fibers of thoracic ascending aortas of WT and mgR mice at age of PD7, PD28, PD35, and PD56. 40× and 100×. b) Gross images of mouse thoracic aortas and hearts at 5 week old WT and mgR mice. Arrow and asterisk indicate the aneurysm in the ascending thoracic aorta of mgR mouse.

**Fig 3:**
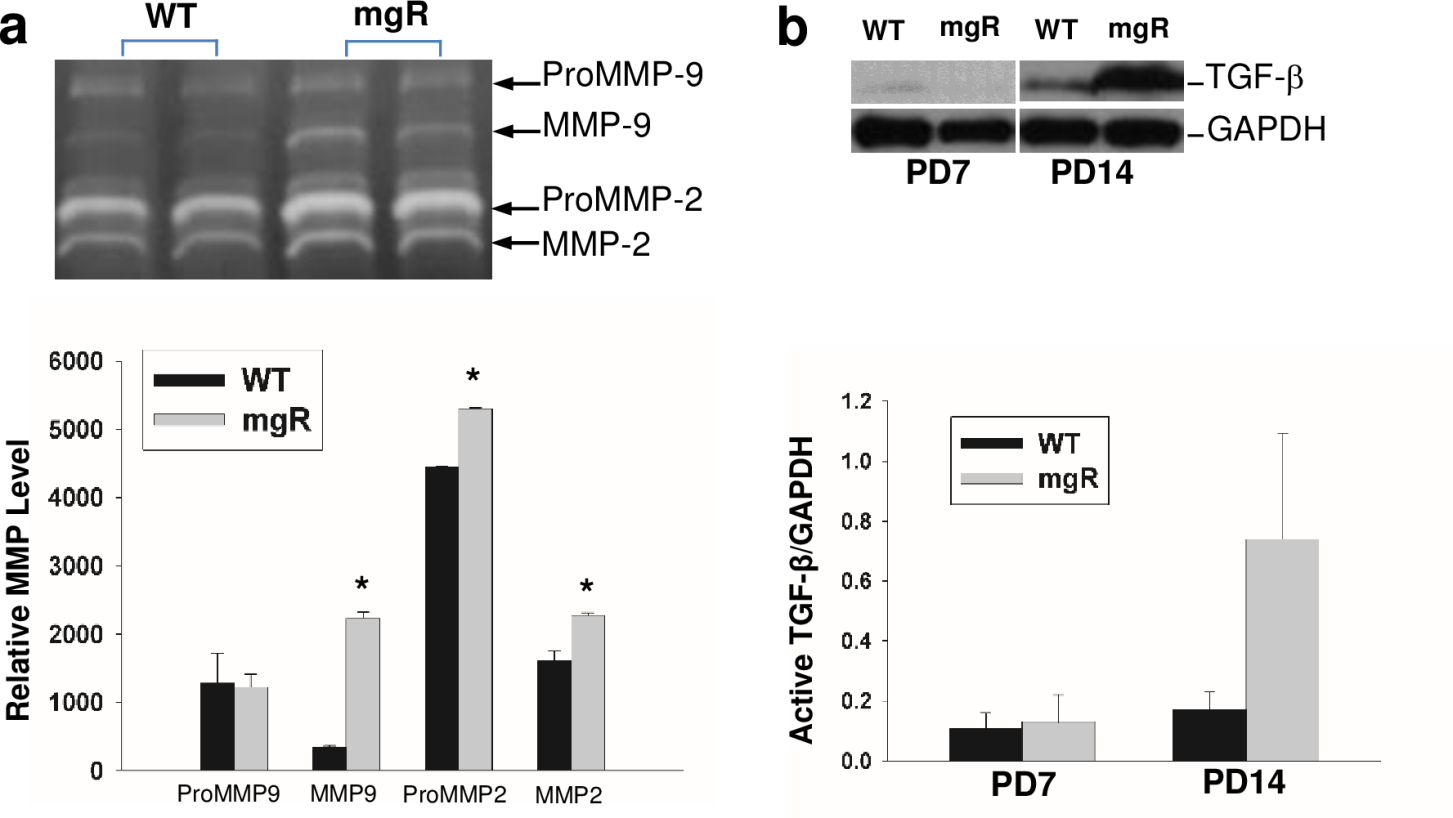
MMP and TGF-β expression in WT and mgR mice. a) MMP-2 and MMP-9 expression in the aorta of WT and mgR mice at PD7. MMP-2 and MMP-9 expression in the aortas of WT and mgR mice at PD7 were analyzed by zymography (upper panel). Relative MMP levels shown in bar graph are quantified using 5 or more aortic specimens per group. b) Active TGF-b levels in the aortic tissue of WT and mgR mice. Aortic TGF-b levels in WT and mgR mice were analyzed by Western blot (upper panel); Representative Western blot analysis of active TGF-b in the aorta of WT and mgR mice (bar graphs represent 4–6/group; mean ± SEM). *p<0.05.

The aortic elastic lamellae are disrupted on PD7 in mgR mice and progressively worsen with age. Disruption of the orderly elastic lamellae, progressive dilatation, and rupture represent the natural history of MFS in patients without surgical intervention. The mgR mice demonstrate the same progression of events. In previous studies we found that doxycycline and losartan were able to inhibit aneurysmal dilatation or rupture when treatment was begun immediately after birth, while they had no effect on lifespan in WT littermates [6]. This was accomplished by treatment through the mother’s milk beginning on PD1[20]. In the present study, we examined the impact of beginning treatment in WT and mgR mice on PD21 (Fig 1). There was no effect on the time to rupture when therapy was started at this time point. This suggested that important changes were occurring in the early (PD0-PD21) postnatal period. We examined histological changes at PD7, a critical time in normal aortic development in the mouse (19). Aortic elastic lamellae in mgR mice showed disorganization and fragmentation and mgR aortas were easily distinguishable from WT littermates at this age (Fig 2A). Our ultrasound and external aorta images indicate thoracic aortic aneurysm formation begins at PD35 (Fig 2B). We further examined the histological changes on PD28, PD35 and PD56. Medial hypertrophy and fragmentation of elastic fibers progress with age (Fig 2A). We also examined MMP levels in mouse aortas. MMP-2 and -9 levels in mgR mouse aortas were significantly increased compared to WT controls on PD7 (Fig 3A). These results demonstrate early metabolic changes in the aorta and suggest that there may be fundamental differences in SMC phenotype as early as PD7.

### TGF-β activity is not increased on PD7

Many studies have implicated enhanced TGF-β activation and signaling in aneurysm formation in MFS [13, 24]. TGF- β would be expected to lead to significant changes in SMC gene expression. We measured active TGF-β levels in the aortas of mgR and littermate controls on PD7 and PD14. As shown in Fig 3B, at PD7, active TGF-β levels in mgR mice were not increased. By PD14 there was a trend toward increased levels of active TGF-β. These data demonstrate that these early histological changes apparent by PD7 in the aorta of mgR mice are not due to increased activation of TGF-β.

### Aortic SMC phenotype switching in mgR mice during early development results in reduction of elastin synthesis

**Fig 4:**
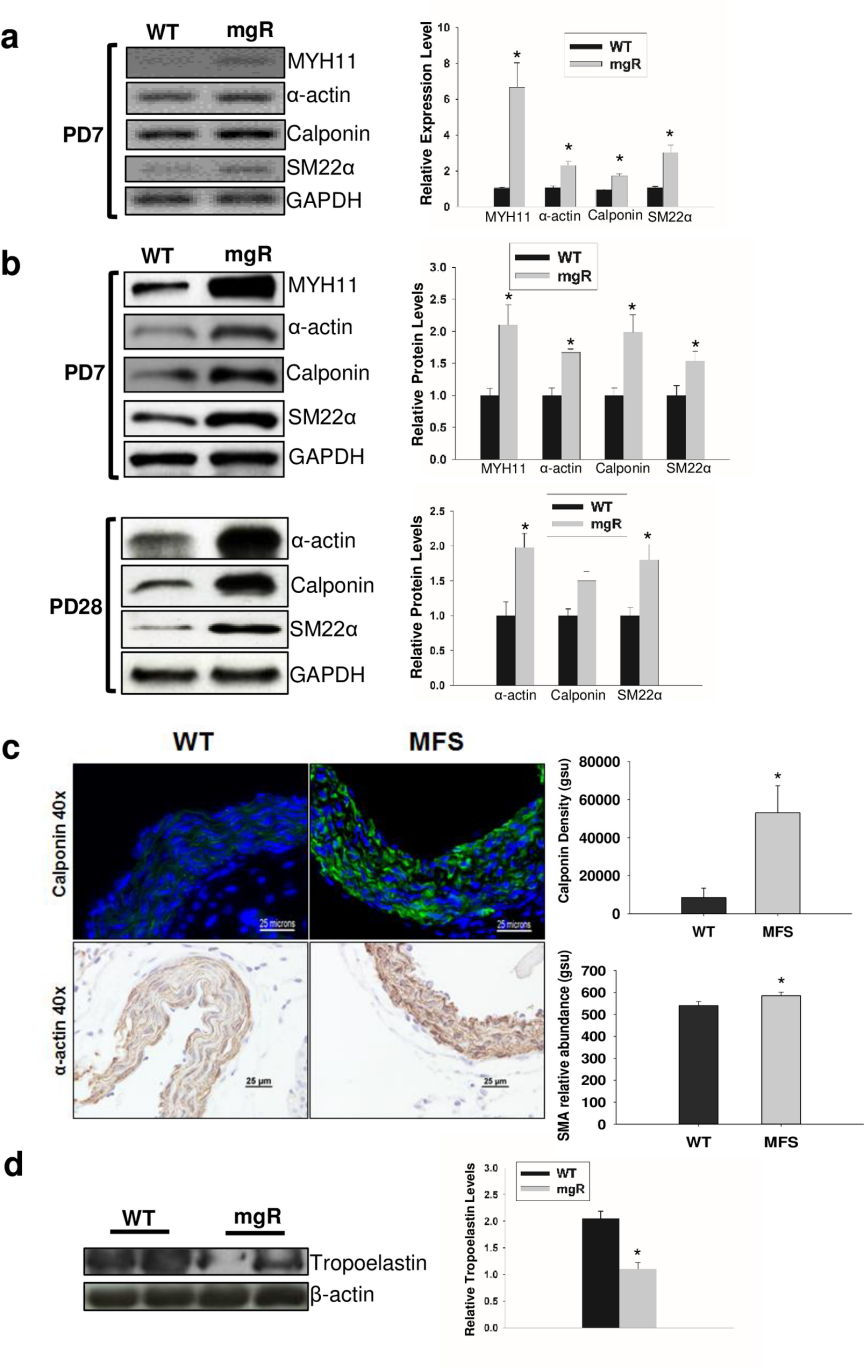
SMC phenotype between WT and mgR mice. a) Aortic SMC contractile marker mRNA expression levels in WT and mgR mice at PD7. Right panel shows relative mRNA levels quantified using 8 aortic samples in each group; b) Aortic SMC contractile protein levels in WT and mgR mouse aortas at PD7 and PD28 analyzed by Western blot. GAPDH served as internal loading control; c) Top panel, Immunofluorescence images are representative staining of calponin in aortic tissue from WT and mgR at PD7; Bottom panel, representative images of immunohistochemical staining for α-actin in aortic tissue from WT and mgR at PD7. Right panel, bar graph depicts the quantification of calponin and α-actin staining. *p<0.05. d) Aortic SMC tropoelastin expression in WT and mgR mouse aortas at PD7 analyzed by Western blot. Western blots represent 3–5 separate experiments. Protein levels were quantified and shown in bar graphs (b & d right panels). β-actin served as internal loading control. Data are represented as mean ±standard error of mean. Significance denoted as *p<0.05.

VSMCs may exhibit two distinct phenotypes, contractile or synthetic. The synthetic phenotype is typically associated with vasculogenesis, growth, and repair. The contractile VSMC phenotype, seen in the normal adult aorta, is characterized by high levels of contractile gene expression, including α-actin, calponin, and SM22α, and low rates of proliferation and extracellular matrix synthesis. The matrix changes seen on PD7 suggest changes in the SMC phenotype. The SMC phenotype was analyzed in mgR mice and WT control mice using RT-PCR. The expression levels of a variety of markers associated with the contractile phenotype, including SM-myosin heavy chain (MYH11), α-actin, calponin, and SM22α were all significantly higher in mgR mouse aortas than in WT control mice (Fig 4A). Changes in these same VSMC contractile proteins in WT and mgR mouse aortas were evaluated by Western blot (Fig 4B). This demonstrated a corresponding increase in contractile protein levels in mgR mouse aortas compared with WT controls. Immunostaining for calponin and α-actin corroborated these results (Fig 4C). We then wanted to know if this SMC phenotype switching affects elastin synthesis during this critical time in aortic development as the high levels of contractile protein markers seen on PD7 suggested that the aortic SMC in MFS were prematurely differentiating into a contractile phenotype. We were particularly interested in tropoelastin expression. Unlike other matrix macromolecules in the aorta, tropoelastin production occurs mainly in the late prenatal and early postnatal time period (19). This late surge in tropoelastin accounts for a significant proportion of the total elastin. We examined the tropoelastin levels in the aortas by Western blot. Tropoelastin levels were decreased by one third in the aorta of mgR mice compared to WT littermates (Fig 4D).

### Aortic SMC proliferation is decreased in mgR mice

**Fig 5:**
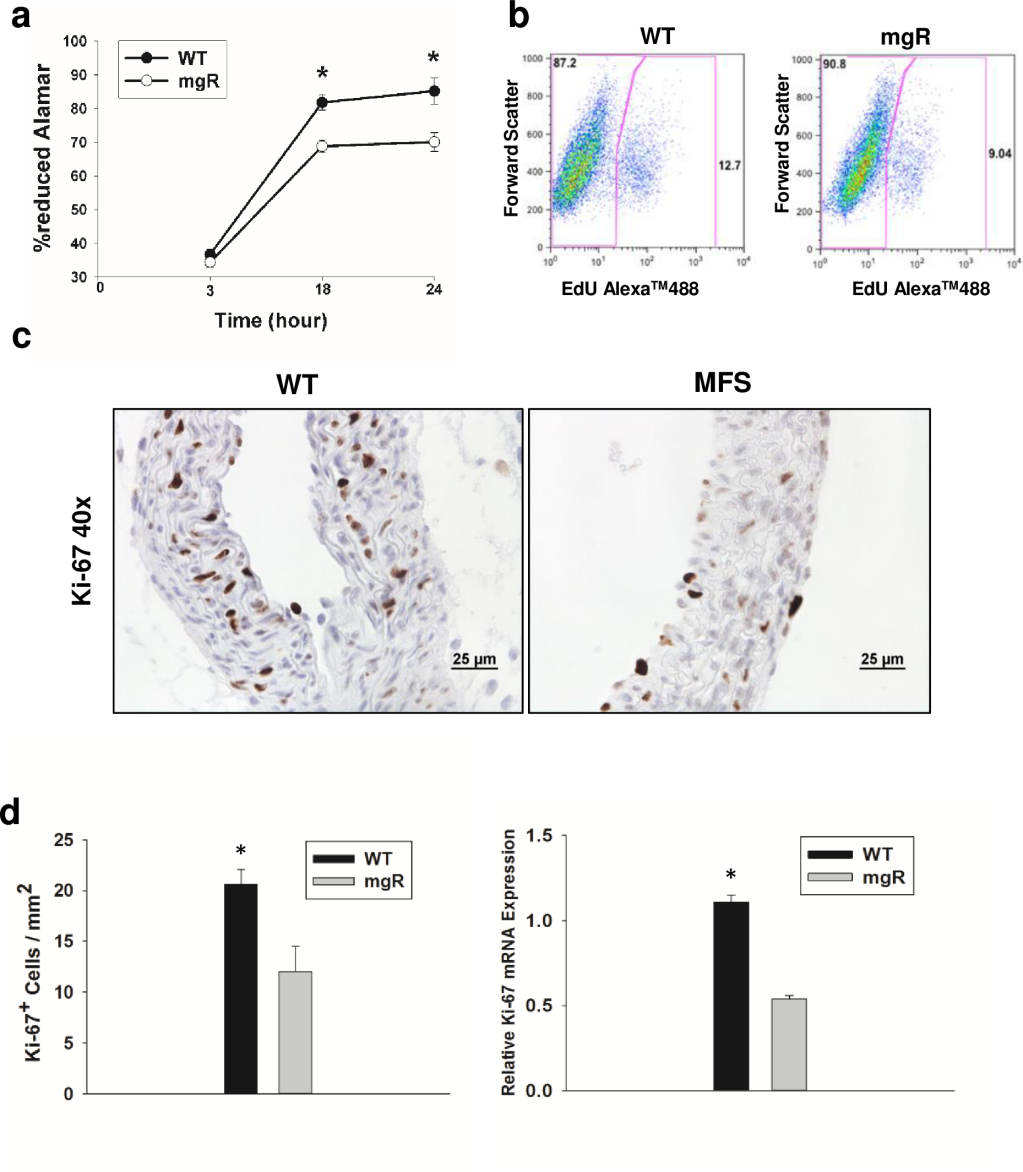
Proliferation of cultured mgR and WT SMCs. a) Aortic wild-type SMCs (n = 3) and mgR SMCs (n = 3) were plated at a density of 2500 cells/well in triplicate. After cell attachment, AlamarBlue was added and its reduction quantified in SMCs at 3, 18, and 24 hours. Data are presented as mean ±SE. Comparisons between groups were done by ANOVA. *p<0.05. b) FACS analysis of aortic SMC proliferation from WT and mgR mice using EdU. c) Representative images of immunohistochemical staining for Ki-67 in WT and mgR aortic sections from PD7 mice. d) Left panel, positive Ki-67 cells were counted and normalized to total cells per area (mm^2^). Right panel, Real-time qPCR mRNA expression for Ki-67 in WT and mgR mouse aortas. *p<0.05.

In addition to high levels of matrix production, aortic SMC proliferation is important to normal aortic development. To examine whether the decrease in fibrillin-1 expression in mgR mice impacts SMC proliferation, the growth curves of early passage (P2) *ex vivo* aortic SMCs from mgR and WT mice were assessed using an AlamarBlue assay. The proliferation rate, obtained for the time points indicated in Fig 5A, was decreased in cells from the mgR aorta. These results were further corroborated using a flow cytometry cell proliferation assay (Fig 5B). This demonstrated a lower percentage of cells from the Marfan aorta were undergoing replication (9.0%) compared to cells from the WT mouse aorta (12.7%). To further extend these proliferation results we performed immunohistochemical staining in aortic sections for the proliferation marker Ki-67 in WT and mgR mice (Fig 5C). The ratio of Ki-67 positive cells to total SMCs in a defined area (mm^2^) were quantified and graphed in Fig 5D, left panel, indicating less proliferating SMCs in mgR mice compared to WT littermates. In addition we performed real-time qPCR for Ki-67 on isolated WT and mgR aortas (n = 3 per group). Aortic Ki-67 mRNA expression were significantly lower in mgR mice compared to WT mice at PD7 (Fig 5D, right panel). Taken together, these findings demonstrate that a lower expression of normal fibrillin-1 in mgR mice leads to decreased aortic SMC proliferation, a finding consistent with SMC polarization toward a more contractile state.

### Abnormal aortic SMC phenotype switching in mgR mice is associated with downregulation of KLF4

**Fig 6:**
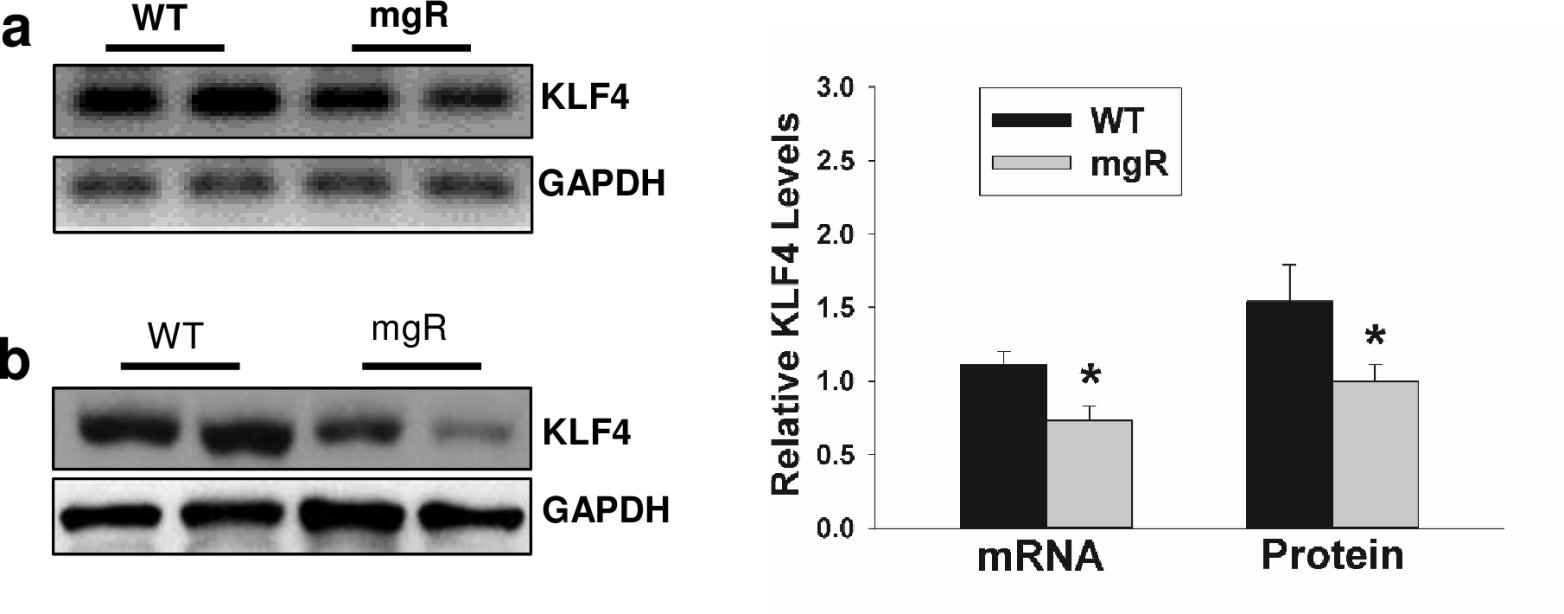
KLF4 expression in mgR and WT mice. a) Aortic KLF4 mRNA expression (a) and protein (b) levels in WT and mgR mice at PD7 were analyzed by RT-PCR (a) and Western blot (b). Gels are representatives of 3 separate experiments. Relative mRNA and protein levels were quantified using 5–8 aortic samples in each group. GAPHD served as internal loading control, *p<0.05.

Expression of VSMC contractile genes is regulated by a variety of factors [25, 26]. Negative regulators such as KLF4, or positive regulators such as TGF-β and BMP4, control transcription of contractile proteins. KLF4 antagonizes contractile gene expression. Therefore, we examined KLF4 mRNA and protein expression in mouse aortas on PD7 by RT-PCR and Western blot, respectively. Both KLF4 mRNA and protein levels were significantly decreased in mgR mouse aortas compared to WT controls (Fig 6A & 6B).

### Re-expression of KLF4 in aortic mgR SMC normalizes expression of contractile markers and proliferation capacity

**Fig 7:**
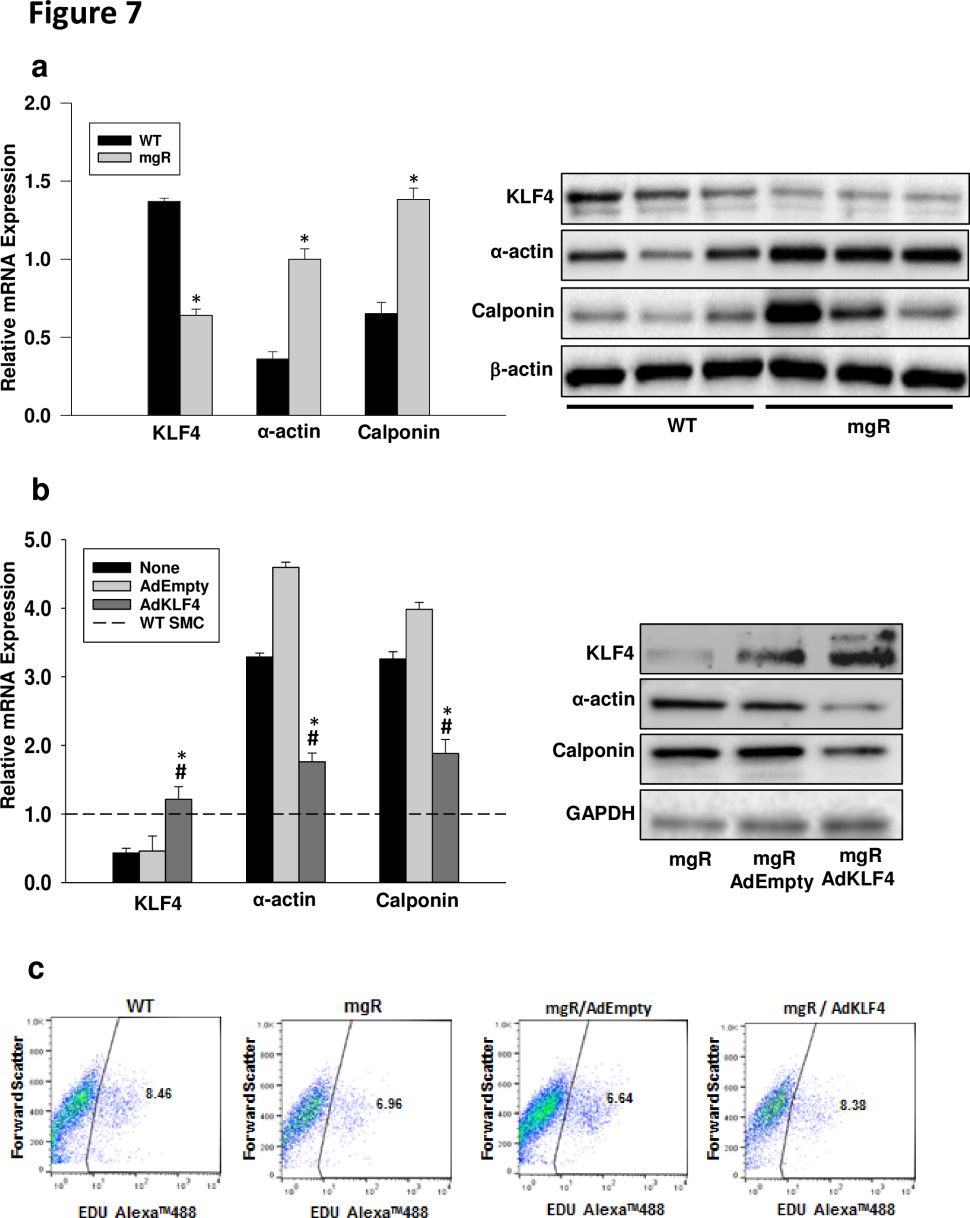
Overexpression of KLF4 in mgR SMCs normalized expression of contractile markers. a) KLF4 mRNA levels were lower in the mgR SMCs compared to WT SMCs derived from PD7 aortic tissue. Both α-actin and calponin mRNA were significantly higher in mgR SMCs compared to WT SMCs. Values represent the mean relative qualification of three cell lines per group (*p< 0.05 WT vs. mgR). This was consistent with difference in protein expression shown by Western Blot (right panel). b) Bar graph demonstrates KLF4, α-actin, and calponin mRNA expression after adenoviral transduction of mgR SMCs. Dotted line represents WT SMC expression. Adenoviral transduction with KLF4 expressing vector resulted in increased KLF4 and decreased α-actin and calponin expression (mean ± SEM, n = 3. * p < 0.05 vs. no infection and # p < 0.05 vs. AdEmpty control infection. 18s was used to normalize samples. Right panel represents a Western Blot showing downregulation of α-actin, and calponin protein expression after adenoviral KLF4 overexpression. c) FACS analysis of aortic SMC proliferation using EdU. Aortic SMCs were isolated from WT and mgR mice. SMCs from mgR mice were infected with adenovirus containing KLF4 (AdKLF4). As a negative control, SMCs from mgR mice were infected with empty vector adenovirus (AdEmpty). Cells from WT and mgR mice without adenoviral infection were baseline controls.

To confirm the role of KLF4 in aortic SMC phenotype in MFS, aortic SMCs were isolated from WT and mgR mice. Consistent with the results from mouse aortas, KLF4 levels in SMCs were significantly lower in mgR mice than WT mice (Fig 7A). Concurrently, SMCs from mgR mice expressed higher levels of contractile proteins, including α-actin and calponin (Fig 7A). Furthermore, we investigated whether a decrease in KLF4 expression was, in part, responsible for the SMC phenotypic switch in mgR mice. SMCs from mgR mice were infected with an adenovirus containing a KLF4 expression vector (AdKLF4). The overexpression of KLF4 by the AdKLF4 transduction was confirmed by Western blot and real-time RT-PCR (Fig 7B). Adenovirus with an empty vector was used as a negative control (AdEmpty). Overexpression of KLF4 reduced the expression of contractile markers, α-actin and calponin, compared to the AdEmpty control (Fig 7B). Next we examined whether overexpression of KLF4 could normalize the proliferation rate of mgR SMCs towards that of the WT SMCs. Using the EdU flow cytometry cell proliferation assay, we found that overexpression of KLF4 with adenovirus (AdKLF4) exhibited a higher percentage of mgR SMCs undergoing replication (8.36%) compared to both no viral infection (mgR) or an empty vector control (AdEmpty) (6.96 and 6.64, respectively). The replication rate after KLF4 overexpression is similar to that of WT aortic SMCs (8.46%) (Fig 7C). These findings demonstrate the reversibility of early smooth muscle cell differentiation markers found in the mgR mice and suggest that KLF4 overexpression could play a role in preventing the early developmental of abnormalities that occur in Marfan aorta.

## Discussion

Thoracic aortic aneurysm and/or aortic dissection (TAAD) are the most deadly manifestation of the inherited connective tissue disorder, Marfan syndrome. Weakness and progressive wear of the aortic matrix was the presumed cause when the linkage between MFS and fibrillin-1 was established [27]. Subsequent work using a variety of genetically engineered murine models of MFS suggested that this structural problem was exacerbated by important metabolic changes in matrix metabolism related to increased TGF-β activity. Antagonism of TGF-β by antibodies prevented the pulmonary manifestations of the disease (13). We have subsequently shown that doxycycline, which prevents release of active TGF-β through inhibition of MMP-2, delays aneurysm formation and rupture in murine models of MFS. Addition of losartan enhanced this activity. Finding that these therapies were effective if begun on PD1 yet ineffective when started on PD21, led us to investigate the aorta during a critical and well-characterized period of development, PD7. Our results demonstrate that aortic SMC in a relatively severe and well-characterized model of MFS, the mgR mouse, prematurely differentiate into a contractile phenotype, a change that would be expected to have important implications for disease progression and, potentially, for therapy.

Given the ineffectiveness of therapy begun as early as PD21, we compared the aorta in mgR and WT littermates at PD7, PD28, and PD56. Aneurysm formation is grossly apparent at PD35. A previous study indicated that newborn homozygous mgR mice showed no phenotypic or histological differences when compared to heterozygous mgR mice [28]. However, histologically, the mgR aorta exhibits clear differences compared to WT mice on PD7; most notable is the elastin fragmentation and disorganization. These findings are consistent with previous EM studies by Bunton et al., which demonstrated a smooth appearance of the elastin fibers in mgR while elastin fibers from the WT mice show regularly spaced protrusions [29]. Given the known role of fibrillin-1 in SMC attachment, these projections might be assumed to be involved in SMC interactions with the matrix. We were interested to know if these changes in the elastin fibers seen on PD7 were perhaps mediated by increased levels of active TGF-β in response to the altered SMC-matrix interaction. By Western blot, we found that active TGF-β levels did not differ between WT and mgR mice at PD7; there is a trend toward increased active TGF-β levels at PD14. Our results are consistant with the work by Cook, et. al. who reported that the intitial consequence of fibrillin-1 mutation was not a significant increase in TGF-β signaling. They demonstrated that inhibition of TGF-β at the early time point of PD16 in the mgR mice exacerbated TAA formation and showed a contrasting beneficial effect when administered at PD45 [30]. However, in agreement with our studies, the early losartan treatment significantly improved survival of mgR mice [6]. The dimorphic effects of TGF-β, particulary that early inhibition exacerbates TAA formation, provides an increased rationale for this study to identify new biomarkers and therapies independent of the TGF-β signaling pathway, such as KLF4, to better understand the early developmental changes in Marfan’s Syndrome.

The increase in active TGF-β levels seen at later time points in the Marfan aorta has been linked to the inability of the matrix to sequester and maintain TGF-β in a latent state. This is associated with decreased binding of TGF-β to the latent TGF-β binding proteins (LTBP) [31]. LTBPs are a family of secreted multi-domain proteins that interact with TGF-β. The binding of TGF-β to the LTBP is by a disulfide bond, which allows it to remain inactive by preventing it from binding to its receptors. MMP-2 allows the release of active TGF-β. Interestingly, MMP-2 levels were increased in Marfan mice at PD7 compared to their WT littermates. Since the activation of TGF-β in the Marfan aorta occurs through its release from the matrix by MMP-2, the increased MMP-2 preceding increases in active TGF-β suggests that the altered SMC phenotype may, in fact, result in the subsequent increases in activated TGF-β.

Neuroectodermally-derived SMCs are the predominant cells populating the media of the ascending aorta. During aortic development, the SMCs must be capable of performing a variety of diverse functions in a precise sequence and timeframe including proliferation, migration, and matrix production. This appears to be especially true for the components of the elastic lamellae since the ability to synthesize, in correct proportions, these matrix macromolecules including fibulin, fibrinogen, fibrillin, and tropoelastin appears restricted to the prenatal and early postnatal period [32]. Subsequently, through post-transcriptional regulation of tropoelastin mRNA, the aorta appears to lose its ability for the coordinated synthesis of proteins required to make normal elastin fibers. The early changes we observe at PD7 in the elastin lamellae led us to assess the phenotype of the aortic VSMC at that age. The proliferative, synthetic SMC phenotype, associated with relatively low levels of expression of SMC contractile proteins such as α-actin, myosin heavy chain, calponin, and SM22α occurs during normal development. As anticipated, this is the phenotype observed in WT littermates at PD7. This contrasted with the contractile phenotype in the mgR aortic tissue associated with increased levels of MYH11, α-actin, SM22α, and calponin. Fully differentiated SMCs in mature blood vessels produce only small amounts of extracellular matrix proteins. This demonstration that aortic SMC in mgR mice switch prematurely to a more mature, contractile phenotype during early development has important implications that not only account for the early matrix abnormalities seen in mice with Marfan syndrome, but have far reaching implications for progression of the aortic pathology.

Elastin is the most abundant protein in the aorta and, in combination with other connective tissue proteins, gives the aorta its viscoelastic property. There is a rapid increase in tropoelastin expression in the late fetal and early postnatal period of aorta [33], which is essential for elastic lamellar unit formation in the aortic wall. This occurs in association with increases in both wall stress and blood pressure. Lamellar units are established early in development and lamellar number is linearly related to wall tension across a broad range of mammalian species [19]. Given the premature differentiation of the aortic SMCs found in the mgR mice, we assessed tropoelastin levels in the aorta at PD7. These levels are decreased by one third in the mgR aorta. This may contribute to the thinner lamellae and elastin fragmentation observed in mgR mice prior to aneurysm formation. This finding was in contrast to past reported results showing upregulation of aortic tropoelastin mRNA expression in 10–14 month old mgR mice [29]. This distinction may be due to the age of the mice. Upregulation of TGF-β in adult mgR mice may stabilize tropoelastin mRNA [34] [35]. These changes in relative tropoelastin levels may reflect the true progression of a dysregulated aortic elastogenesis process in Marfan’s Disease.

The plasticity of the SMC, as it moves between a synthetic and contractile phenotype, is critical not only for normal development, but also for a normal response to injury. Expression of VSMC contractile genes is regulated by a variety of factors [25, 26]. KLF4 is known to be a master regulator of SMC phenotype; it antagonizes contractile gene expression. Both KLF4 mRNA and protein levels were significantly decreased in mgR mouse aortas compared to WT controls. This is consistent with the increase in contractile markers in mgR mice. In order to better understand the role of KLF4 and to determine if these changes in mgR SMC were reversible, we overexpressed KLF4 in mgR SMCs. This resulted in normalization of both *in vitro* contractile markers and proliferation rate. Considering the detrimental effects of the early phenotype switch seen mgR mice, preventing this by *in vivo* overexpression of KLF4 has the potential to inhibit the early developmental abnormalities that occur in the Marfan aorta and this will be the focus of our future work.

## Conclusion

In summary, our data establish that decreased fibrillin-1 expression is associated with an intrinsic defect in aortic SMCs. The premature switch from a synthetic to a contractile phenotype results in decreased elastin synthesis during early aortogenesis. Impaired proliferation of VSMCs and altered matrix metabolism would be expected to further exacerbate the matrix abnormalities in the aortic wall. This early phenotypic switch would explain why losartan and doxycycline were ineffective if started at PD21 compared to at PD0. Furthermore, our findings are consistent with clinical data suggesting that pharmacological intervention is more effective when therapy is initiated in younger patients compared to adults [36]. In summary, we have identified a novel therapeutic target in the early pathogenesis of Marfan syndrome that may significantly impact the course of the disease. Future studies will assess the effects of blocking this early phenotypic switch on subsequent aortic development and aneurysm formation.
