# Correction: Premature aortic smooth muscle cell differentiation contributes to matrix dysregulation in Marfan Syndrome

**DOI:** 10.1371/journal.pone.0200985

**Published:** 2018-07-16

**Authors:** Matthew Dale, Matthew P. Fitzgerald, Zhibo Liu, Trevor Meisinger, Andrew Karpisek, Laura N. Purcell, Jeffrey S. Carson, Paul Harding, Haili Lang, Panagiotis Koutakis, Melissa Suh, Rishi Batra, Constance J. Mietus, George Casale, Iraklis Pipinos, B. Timothy Baxter, Wanfen Xiong

Dr. Melissa Suh is not included in the author byline. She should be listed as the twelfth author and her affiliation is 1: Department of Surgery, University of Nebraska Medical Center, Omaha, Nebraska, United States of America. The contributions of this author are as follows: Data Curation, Methodology, Writing–Original Draft Preparation, and Writing–Review & Editing.
